# Molecular docking analysis of FDA approved drugs with the glycoprotein from Junin and Machupo viruses

**DOI:** 10.6026/97320630018119

**Published:** 2022-02-28

**Authors:** Himani Malhotra, Arvind Kumar, Yasir Afaq

**Affiliations:** 1 Department of Biotechnology, Lovely Professional University, Jalandhar-Delhi G.T. Road, Phagwara, Punjab, INDIA - 144411; 2 Department of Computer Science, Lovely Professional University, Jalandhar-Delhi G.T. Road, Phagwara, Punjab, INDIA - 144411; 3 Department of Biochemistry, Lovely Professional University, Jalandhar-Delhi G.T. Road, Phagwara, Punjab, INDIA - 144411

**Keywords:** Drug design, ligands, Junin virus, Machupo virus, glycoprotein

## Abstract

Arenaviruses, Junin and Machupo are pathogenic viruses in regions of South America including Argentina and Bolivia causing haemorrhagic fever among humans. They have been transmitted to humans through mouse causing chronic illness with high mortality. Therefore, it is of interest to acquittance the molecular docking analysis data of FDA approved drugs with the glycoprotein from Junin and Machupo viruses for consideration in drug discovery. Thus, we report the optimal binding features of MK-3207 and Dihydro ergotamine with the protein target for further validation and consideration.

## Background:

Junin and Machupo human pathogenic New World Arenaviruses belongs to Mammareavirus genus of Arenaviridae family and were isolated in 1958 from regions of Argentina and Bolivia [1-2]. Junin virus was transmitted to humans from natural occurring reservoirs mainly Calomys musculin and Machupo virus from Calomys callosus [3 & 4]. Symptoms such as frailty, anorectic, pain and fever persuade by incubation of 7-14 days followed by further neurological, constitutional, cardiovascular and gastrointestinal signs [5-6].

The genome of Arenaviruses possessing negative sense single-stranded RNA encompasses two segments pertaining Small (S) RNA segment (3.4 kb) and Large (L) segment (7.2 kb) [7]. Small segment encodes for envelope glycoprotein precursor (GPC) and the nucleoprotein (NP), whereas large segment encodes for matrix protein (Z) and viral RNA-dependent RNA polymerase (L) [7]. The glycoprotein precursor further degraded into N-terminal GP1; having binding capacity with host receptor and the transmembrane GP2, indulge in viral fusion by signal peptidases and subtilisin kexin isozyme-1or site-1 protease (SKI-1/S1P) [7,8 and 4]. Junin and Machupo viruses share a common receptor hTfR1 (human transferring receptor1) [9]. Apart from pervasion of studies for identification of therapeutic facilities for prevention and cure of both viruses, no drug out of date being administered [10]. Computational drug designing approaches are used to predict and evaluate drugs for various endemic (other diseases too) diseases [11,12]. It has reduced the time span of effective and precise drug development. Considerable progress has been made in the areas of drug development pertaining to viral pathogenesis [13]. However, high mutability rates and variable genome dynamics of viruses have been the major obstacles in effective drug design against the detrimental pathogens [14]. With the increase in prevailing threat of Junin and Machupo viruses, there is a rising demand to design drugs for them [15]. Ribavirin (1-D ribofuranosyl.1.2.4. triazole-3-carboxamide) is the only anti-arenaviral drug currently available against Junin virus while it fails to increase the survival benefits among patients and also display many side effects including anaemia and febrile syndrome [10]. Scarcity of effective drugs against the menacing Arenaviruses is another domain of viral genomics that needs to be catered [15,12]. Therefore, it is of interest to document the molecular docking analysis data of FDA approved drugs with the glycoprotein from Junin and Machupo viruses for consideration in drug discovery.

## Materials and methods:

### Retrieval and pre-processing of protein structures:

GP1 subunit of glycoprotein binds to the human receptor transferrin receptor 1 (TfR1) and causes infection among humans [9]. So, highly resolved X-ray diffraction crystal structure of Glycoprotein (GP1) of Junin [9] (Table 1 and Figure 1 - see PDF) and Machupo virus [9] (Table 1 and Figure 2 - see PDF) was retrieved as target from Protein Data Bank (PDB) database. Further refinement of both structures was performed by removal of water molecules, addition of polar hydrogen and Kollaman charges in AutoDock tools [16]. Also, grid box was defined for GP1 of Junin and Machupo viruses within their active site which was concluded using CASTp (Computer Atlas of Surface Topography of Proteins) server [17].

### Retrieval of ligand structures:

Further ligand compounds were retrieved from ZINC15 database [18] by downloading 2115 FDA-approved drugs and 3754 investigational drugs in mol2 format. In addition, compounds prevailing mol2 structures were converted to PDBQT format structures by using Open Babel tool [19] and further PRODRG server [20] was used for energy minimization of the structures.

### Molecular Docking:

Screening of downloaded structures of ligands (.PDBQT format) was performed by computing docked score of each ligand within active site of GP1 target protein from Junin and Machupo viruses separately in AutoDock vina software [21]. Best scored ligands were selected for further analysis [12]. Furthermore, interactions between ligand and target was investigated by using Pymol and PLIP (protein-ligand interactor profiler) [22] tools.

### Drug Likeliness and potential toxicity prediction:

Drug Likeliness [23,24] based on physiochemical properties of best selected ligands were computed using SwissADME [25] and pkCSM servers [26]. The Pan-Assay Interference Structures (PAINS) analysis [27] was also performed in Swiss ADME server for each selected ligand. The toxicity parameters like mutagenicity, carcinogenecity and cytotoxicity of the selected ligands was estimated using the ProTox-II web-server [28] and further validation was performed with the vNN-ADMET server [29].

## Results and Discussion:

Molecular docking of all ligands was performed separately within active site of target structure of Junin virus (5W1K) (Figure 1 - see PDF) and Machupo virus (5W1M) (Figure 2 - see PDF). Active site of 5W1K (Junin virus) target structure was selected by defining grid box dimensions as centre_X=-37.414, centre_Y=-0.048, centre_Z=-85.385; size_x=126, size_y=126, size_z=126 and similarly active site of 5W1M (Machupo virus) target structure was selected by defining grid dimensions as center_X=75.663, center_Y=222.274, center_Z=221.976; size_x=126, size_y=104, size_z=126 in AutoDock Vina software. Binding energy score of each ligand was computed with both target structures separately showing best scored ligands from FDA approved drugs library (Table 2, 8 -see PDF) and from investigational drug library (Table 5, 11 - see PDF). Interactions of ligand with target structures were visualized in Pymol visualization tool [30] as shown in Figure 3 and 4(see PDF). Physiochemical properties based on Lipinski's Rule of Five [31] which includes the following criteria that Molecular weight must be less than 500, Number of hydrogen-bond donors less than 5, Number of hydrogen bond acceptors less than 10 and Log P value must be less than 5 were computed for best scored ligands. These properties help in evaluation of drug-likeliness of ligand structures [32].

Analysis of best hit ligands from FDA-approved drug library (Table 2 - see PDF) [33] and from investigational-drug library (Table 5 - see PDF) for Junin virus showed that only compound ZINC000011679756 with docking score of -8.4kcal/mol (Table 3 - see PDF) follow the Lipinski's rule of five (Table 3 and 6 - see PDF). However, ligands showing mild variations in physiochemical properties (Table 3 and 6 - see PDF) yet can also be considered as modifications in physiochemical properties is also one of the techniques to increase the bioavailability of drug [34,35]. Further, in silico evaluation of toxic parameters [36, 34] was also performed on best selected ligands and compounds active for toxic parameters were not considered further (Table 4 and 7 - see PDF). One of the other parameter Pan-assay interference structures (PAINS), that include fluorescence of small molecules, redox reactivity and covalent modifications of target protein was also evaluated. Only one ligand compound ZINC000011679756 was predicted to possess PAINS value 1 (Table 4 and 7 - see PDF) and was not considered further. Thorough analysis of interaction, physiochemical properties and toxicity predicts ligand with Zinc ID ZINC000043203371 (MK-3207) [37] and docking score -8.6kcal/mol [38] as safe and best candidate for further studies against GP1 protein of Junin virus(Table 5, 6 and 7 - see PDF). This predicts MK-3207 as potent inhibitor for Glycoprotein of Junin virus (Table 14 - see PDF) [39]. Similarly, extensive analysis of physiochemical properties of best docked ligands for Machupo virus was also done which predicts only compound ZINC000066166864 with docking score of -10kcal/mol (Table 8 - see PDF) has drug-likeliness according to Lipinski's rule of Five (Table 9 - see PDF). Other ligands showing mild variations in physiochemical properties (Table 9 and 12 - see PDF) can also be considered as potent drugs. Ligands showing violations in more than 2 rules are considered to be of low solubility or permeability and cannot be preceded further [40,41]. Toxicity parameters was also analysed to eradicate toxic compounds (Table 10 and 13 - see PDF). Thorough analysis of physiochemical properties and toxicity predicts ZINC000003978005 (Dihydroergotamine) [42] with docking score -11kcal/mol as safe and best candidate for further in vitro and in vivo studies to predict it as potent drug for GP1 protein of Machupo virus (Table 14 - see PDF).

## Conclusion:

We report MK-3207 and Dihydroergotamine with optimal binding features as potent inhibitors of glycoprotein in Junin and Machupo viruses and can be considered further for validation.
